# Maternal Serum Folate During Pregnancy and Congenital Heart Disease in Offspring

**DOI:** 10.1001/jamanetworkopen.2024.38747

**Published:** 2024-10-10

**Authors:** Yanji Qu, Xiaoqing Liu, Shao Lin, Michael S. Bloom, Ximeng Wang, Xiaohong Li, Hui Wang, Fengzhen Han, Ju-E. Liu, Weixiang Pan, Wangjian Zhang, Xia Zou, Jian Zhuang, Jie Li, Jimei Chen

**Affiliations:** 1 Global Health Research Center, Guangdong Provincial People’s Hospital, Guangdong Academy of Medical Sciences, Southern Medical University, Guangzhou, Guangdong, China; 2 Guangdong Cardiovascular Institute, Guangdong Provincial People’s Hospital, Guangdong Academy of Medical Sciences, Southern Medical University, Guangzhou, Guangdong, China; 3 Department of Environmental Health Sciences, University at Albany State University of New York, One University Place, Rensselaer, Albany; 4 Department of Global and Community Health, George Mason University, Fairfax, Virginia; 5 Department of Obstetrics and Gynecology, Guangdong Provincial People’s Hospital, Guangdong Academy of Medical Sciences, Southern Medical University, Guangzhou, Guangdong, China; 6 Department of Pharmacy, Guangdong Provincial People’s Hospital, Guangdong Academy of Medical Sciences, Southern Medical University, Guangzhou, Guangdong, China; 7 Department of Medical Statistics, School of Public Health, Sun Yat-sen University, Guangzhou, China; 8 Department of Epidemiology and Center for Global Cardiometabolic Health, School of Public Health, Brown University, Providence, Rhode Island

## Abstract

**Importance:**

The association of folate supplementation with congenital heart disease (CHD) prevention is controversial.

**Objective:**

To examine the association of maternal serum folate levels at early to midpregnancy with CHD risk in offspring.

**Design, Setting, and Participants:**

This case-control study recruited participants from one of China’s largest cardiac referral centers between 2015 and 2018. CHD cases and non-CHD controls were matched according to maternal age at a ratio of 1:4. Data were analyzed from May to August 2023.

**Exposures:**

Maternal serum levels of folate, vitamin B_12_, and homocysteine were measured around the gestational age of 16 weeks.

**Main Outcomes and Measures:**

The primary outcome was CHD, which was confirmed using echocardiography. The association between CHD risk in offspring with maternal folate levels was measured using adjusted odds ratios (aORs) with 95% CIs in conditional logistic regression analyses. Interactions between folate, vitamin B_12_, and homocysteine and CHD were estimated on a multiplicative scale.

**Results:**

A total of 129 CHD cases with ventricular septal defect as the most common phenotype and 516 matched controls were included. The mean (SD) maternal age at pregnancy was 31.6 (5.3) years. There was a U-shaped association between maternal serum folate levels at early to midpregnancy and CHD risk in offspring. Compared with the offspring in the second and third quartiles of maternal folate, those in the lowest (aOR, 3.09; 95% CI, 1.88-5.08) and highest quartiles (OR, 1.81; 95% CI, 1.07-3.06) had increased odds of CHD. The ORs were higher when applying the World Health Organization criteria to determine the normal range for serum folate levels. Interaction analyses suggested that the adverse associations between low and high maternal folate and CHD risk might be further magnified by vitamin B_12_ deficiency or elevated homocysteine.

**Conclusions and relevance:**

In this case-control study of CHD, low maternal serum folate levels in early to midpregnancy were associated with an increased CHD risk in offspring, and excessively high folate levels were also associated with an elevated CHD risk. Further investigation is needed to make causal inferences for the observed associations and elucidate the underlying mechanisms.

## Key Points

**Question:**

What is the association between maternal serum folate and congenital heart disease (CHD) among offspring?

**Findings:**

In this case-control study of 129 patients with CHD, the association between maternal serum folate levels and CHD risk in offspring was U-shaped; both low and high maternal folate were associated with a higher risk of CHD. Maternal vitamin B_12_ deficiency and homocysteine elevation were relevant factors.

**Meaning:**

These findings suggest that in addition to preventing folate deficiency, it is also important to be aware of the potentially harmful effects of excess folate on CHD in offspring.

## Teaser

This case-control study evaluates the association of maternal serum folate levels at early to midpregnancy with congenital heart disease risk in offspring.

## Introduction

Sufficient folate intake during pregnancy is crucial for preventing neural tube defects (NTDs) in offspring.^1^ Whether folate has the same protective effects on congenital heart disease (CHD) has been widely debated.^2,3,4^ CHD is the most prevalent birth anomaly worldwide, affecting approximately 2.3% of live births,^5^ which is more than 10 times higher than NTDs.^6^ If increasing folate levels could reduce CHD, then the public health significance goes above that of preventing NTDs alone.^6^

Previous studies on the role of folate in preventing CHD have had conflicting findings. A Hungarian randomized controlled trial^7^ in the 1980s showed that a multivitamin supplement with 0.8 mg of folic acid significantly reduced CHD risk. This study inspired observational studies to verify the protective effect of folate on CHD.^8,9,10,11^ About half of these studies found a protective association with folic acid supplementation of at least 0.4 mg per day, while others found no effect. Conversely, a recent meta-analysis^11^ found increased folic acid intake associated with a higher risk of atrial septal defect, a common CHD phenotype. Conflicting findings also exist regarding maternal blood folate levels and CHD risk, with some studies^12,13,14^ reporting a link between lower folate levels and higher CHD risk, while others^15,16,17^ found no association.

With widespread recommendations for folate supplementation and mandatory folic acid fortification in foods, folate intake among women of childbearing age has increased globally.^18,19^ Given the contradictory findings, it is urgent to investigate the relationship between maternal folate status, especially high folate levels, and CHD risk. Folate, along with vitamin B_12_ and homocysteine, is crucial in one-carbon metabolism.^20^ Both folate and vitamin B_12_ are needed to convert homocysteine to methionine; deficiencies in either can lead to elevated homocysteine levels, increasing CHD risk.^21^ Understanding their role in CHD causes will inform preventive nutritional strategies during pregnancy. Therefore, we conducted this large case-control study to examine the dose-response association between maternal serum folate levels at early to midpregnancy and CHD risk in offspring, as well as the joint associations between maternal serum levels of folate, vitamin B_12_, and homocysteine, as well as CHD risk.

## Methods

### Study Design and Participants

We recruited 9540 offspring receiving routine prenatal care from Guangdong Provincial People’s Hospital, a large cardiac referral center in China, between 2015 and 2018. All pregnancies were followed up for delivery outcomes. CHD was diagnosed and confirmed through a comprehensive multistage approach (eMethods in Supplement 1). We conducted a case-control study to assess the association between maternal serum folate levels and CHD risk in offspring. For each CHD case, we selected 4 controls matched by maternal age at pregnancy. Preterm births with simple patent foramen ovale and patent ductus arteriosus, nonsingleton pregnancies, and CHD cases with chromosomal abnormalities, gene mutations, or extracardiac malformations were excluded. Finally, we included 129 CHD cases and 516 matched non-CHD controls (eFigure 1 in Supplement 1). Our study complies with the Declaration of Helsinki and was approved by the ethics committee of Guangdong Provincial People’s Hospital. All pregnant women provided written informed consent. This study followed the Strengthening the Reporting of Observational Studies in Epidemiology (STROBE) reporting guideline.^22^

### Measurement of Maternal Folate Status

Periconceptional folic acid supplementation was self-reported by participants during face-to-face interviews at enrollment and treated as a binary variable (yes or no) in the regression models. The recommended supplement contained 0.8 mg of folic acid per capsule. Serum folate levels were measured to assess folate status more precisely. Serum samples were collected at the first prenatal care visit, around 16 weeks’ gestation, and analyzed using chemiluminescence microparticle immunoassay on the ARCHITECT i1000SR Analyzer (Abbott Laboratories), with an interassay coefficient of variation of 10.7% and an intra-assay coefficient of variation of 4%. Vitamin B_12_ and homocysteine levels were also measured. Serum folate levels were classified into low, medium, and high strata based on 2 criteria: quartiles (lowest quartile as low, second and third quartiles as medium, highest quartile as high) and the World Health Organization (WHO) criteria for macrocytic anemia (deficiency, <5.9 ng/mL; normal, 5.9-20 ng/mL; elevated, >20 ng/mL).^23^

### Covariates

At enrollment, all pregnant women included in the case-control study completed a structured questionnaire capturing periconceptional information on sociodemographic factors, reproductive history, medication usage during pregnancy, environmental factors, lifestyle behaviors, and family history of cardiovascular diseases. Education was dichotomized into completion of senior high school (≥12 years) or not (<12 years). Occupation was reported as employed or not. Both elective and spontaneous abortion experiences were recorded as positive abortion history. Nulliparous status was defined when the current pregnancy was the first parity. Obstetrical complications and in vitro fertilization and embryo transfer status were extracted from the electronic medical records. Folate metabolism-related genetic polymorphisms, such as 5,10-methylenetetrahydrofolate reductase (MTHFR) C677T, MTHFR A1298C, and 5-methyltetrahydrofolate-homocysteine methyltransferase reductase (MTRR) A66G, were genotyped using polymerase chain reaction.

### Statistical Analysis

We employed conditional logistic regression to assess the associations of low and high maternal serum folate levels with CHD risk in offspring, with the medium folate level serving as the reference group. Covariates were adjusted for in multivariable models. Model 1 is unadjusted. In model 2, we adjusted for periconceptional folic acid supplementation, maternal education, occupation, parity, abortion history, pregnancy with diabetes, pregnancy with hypertension, pregnancy with cardiac diseases, infection, and in vitro fertilization and embryo transfer. In model 3, we additionally adjusted for maternal MTHFR 677, MTHFR 1298, and MTRR 66 polymorphisms. All the covariables were complete, except for folate metabolism-related genetic polymorphisms. For the 46 participants (7%) with insufficient DNA samples for genotyping, we imputed missing values using the wild-type genotype. Folate metabolism-related genetic polymorphisms were not associated with serum folate levels (eTable 1 in Supplement 1). Additionally, we conducted a sensitivity analysis excluding these 46 participants to confirm the robustness of our results.

The nonlinear associations between maternal serum folate levels and CHD risk in offspring were examined using conditional logistic regression including a restricted cubic spline term for folate levels with 3 knots,^24^ with adjustment for the covariables in model 3. The nonlinearity *P* value was estimated using a likelihood ratio test.

To investigate whether the associations of maternal serum folate levels with CHD risk in offspring were modified by maternal vitamin B_12_ deficiency and elevated homocysteine, we further adjusted for maternal serum levels of vitamin B_12_ and homocysteine in model 3 and examined their interaction on multiplicative scales. Vitamin B_12_ deficiency was defined as serum levels below 300 pg/mL.^25^ Elevated homocysteine was defined as serum levels higher than 7 μmol/L.^26,27^ The multiplicative interaction was accessed by adding the product terms to model 3.^28^ To assess the joint associations of folate levels with vitamin B_12_ deficiency or elevated homocysteine, participants were classified into 6 groups: low, medium, or high folate and normal or deficient vitamin B_12_ or normal or elevated homocysteine. This categorized variable was included in model 3, with medium folate and normal vitamin B_12_ or normal homocysteine as the reference group.^29^

Considering the mechanism that deficiencies in folate and vitamin B_12_ can lead to elevated levels of homocysteine, we conducted mediation analysis according to the method proposed by Baron and Kenny to assess the mediation effect of elevated homocysteine on the associations between low folate and vitamin B_12_ deficiency and CHD risk.^30^ Two-sided *P* values and 95% CIs were calculated for statistical inference. All analyses were performed with R version 4.3.2 (mainly packages survival, rcssci, interactionR, and mediation) (R Project for Statistical Analysis). Data analysis was conducted from May to August 2023.

## Results

### Characteristics of Participants

We included 129 CHD cases, with ventricular septal defect as the most common phenotype, and 516 matched controls (Table 1). The mean (SD) maternal age at pregnancy was 31.6 (5.3) years. Among the 645 women included in the study, 611 (95%) reported taking periconceptional folic acid supplements. There was no significant difference in the rates of supplementation and serum folate levels between CHD cases and controls (Table 2). However, women who took folic acid supplements exhibited significantly higher serum folate levels compared with those who did not (eTable 2 in Supplement 1). Compared with controls, CHD cases tended to have 90.2 pg/mL (95% CI, 57.7 to 122.7; *P* < .001) lower vitamin B_12_ but 0.2 mg/L (95% CI, 0.2 to 0.3; *P* < .001) higher levels of homocysteine (Table 2).

**Table 1.** Frequencies of Phenotypes Among Cases With Congenital Heart Disease (CHD)

Phenotypes of congenital heart disease	Participants, No. (%) (N = 129)
Ventricular septal defect	26 (20.2)
Coarctation of aorta	20 (15.5)
Other specified CHD^a^	18 (14.0)
Transposition of the great arteries	16 (12.4)
Pulmonary stenosis	12 (9.3)
Atrial septal defect	10 (7.8)
Tetralogy of Fallot	7 (5.4)
Double-outlet right ventricle	5 (3.9)
Total anomalous pulmonary venous connection	4 (3.1)
Tricuspid regurgitation	4 (3.1)
Ebstein anomaly	2 (1.6)
Mitral valve atresia	2 (1.6)
Atrioventricular septal defect	1 (0.8)
Interrupted aortic arch	1 (0.8)
Mitral regurgitation	1 (0.8)

^a^ Other heart and circulatory system anomalies not included in the current table.

**Table 2.** Maternal Characteristics According to Offspring’s Congenital Heart Disease (CHD) Status^a^

Demographic characteristics	Participants, No. (%)		*P* value
	CHD cases (n = 129)	Controls (n = 516)	
Age, mean (SD), y	31.5 (5.45)	31.6 (5.25)	.93
Education, y			
≤12	3 (2.3)	10 (1.9)	.78
>12	126 (97.7)	506 (98.1)	
Unemployed			
Yes	16 (12.5)	42 (8.2)	.13
No	113 (87.5)	474 (91.8)	
Reproductive history			
Nulliparous			
Yes	72 (55.8)	306 (59.3)	.16
No	57 (44.2)	210 (40.7)	
Elective/spontaneous abortion history			
Yes	53 (41.1)	176 (34.1)	.13
No	76 (58.9)	340 (65.9)	
Obstetrical complications			
Prepregnancy body mass index, mean (SD)^b^	20.7 (3.0)	20.8 (3.2)	.82
Pregnancy with diabetes			
Yes	18 (14.0)	103 (20.0)	.11
No	111 (86.0)	413 (80.0)	
Pregnancy with hypertension			
Yes	10 (7.8)	35 (6.8)	.70
No	119 (92.2)	481 (93.2)	
Pregnancy with cardiac disease			
Yes	15 (11.6)	52 (10.1)	.61
No	114 (88.4)	464 (89.9)	
Pregnancy with infection			
Yes	16 (12.4)	50 (9.7)	.37
No	113 (87.6)	466 (90.3)	
IVF-ET			
Yes	6 (4.7)	26 (5.0)	.86
No	123 (95.3)	490 (95.0)	
Medication usage during pregnancy			
Periconceptional folic acid supplementation			
Yes	122 (94.6)	489 (94.8)	.93
No	7 (5.4)	27 (5.2)	
Antibiotics use			
Yes	7 (5.4)	26 (5.0)	.28
No	122 (94.6)	490 (95.0)	
Antimiscarriage medicine uses			
Yes	11 (8.5)	68 (13.2)	.62
No	118 (91.5)	448 (86.8)	
Gestational weeks at blood collection, mean (SD)	16.45 (5.57)	16.85 (2.55)	.51
Serum folate, ng/mL, median (IQR)^c^	16.0 (10.6-18.6)	17.2 (14.4-18.5)	.06
Serum vitamin B_12_, pg/mL, median (IQR)	280 (208-399)	395 (307-512)	<.001
Serum homocysteine, mg/L, median (IQR)	0.9 (0.7-1.2)	0.7 (0.6-0.8)	<.001
Folate metabolism-related gene polymorphisms			
MTHFR 677			
CC	74 (60.7)	255 (53.5)	.43
CT	35 (28.7)	181 (37.9)	
TT	13 (10.7)	41 (8.6)	
MTHFR 1298			
AA	78 (63.9)	275 (57.7)	.43
AC	37 (30.3)	179 (37.5)	
CC	7 (5.7)	23 (4.8)	
MTRR 66			
AA	66 (54.1)	265 (55.6)	.94
AG	48 (39.3)	168 (35.2)	
GG	8 (6.6)	44 (9.2)	

Abbreviations: IVF-ET, in vitro fertilization and embryo transfer; MTHFR, 5,10-methylenetetrahydrofolate reductase; MTRR, 5-methyltetrahydrofolate-homocysteine methyltransferase reductase.

SI Conversion factors: To convert folate to nmol/L, multiply by 2.266; homocysteine to μmol/L, multiply by 7.397; vitamin B_12_ to pmol/L, multiply by 0.7378.

^a^ The comparison of maternal characteristics between CHD cases and non-CHD controls was conducted using univariate conditional logistic regression.

^b^ Calculated as weight in kilograms divided by height in meters squared.

^c^ The *P* value was .06 in the crude model; .08 in the model with adjustment for gene polymorphisms of MTHFR 677, MTHFR 1298, and MTRR 66; and .99 in the model with additional adjustment for periconceptional folic acid supplementation.

### Associations Between Maternal Serum Levels of Folate and CHD Risk in Offspring

We found a U-shaped association between maternal serum folate levels and CHD risk in offspring (overall *P* < .001, *P* for nonlinearity <.001) (Figure). Compared with the medium levels of folate, both the lowest quartile (adjusted OR [aOR], 3.09; 95% CI, 1.88-5.08) and the highest quartile (aOR, 1.81; 95% CI, 1.07-3.06) of maternal serum folate levels were associated with an increased risk of CHD in offspring. Compared with normal levels of folate defined by the WHO criteria (5.9-20.0 ng/ml), folate deficiency (aOR, 18.97; 95% CI, 3.87-93.11) and elevation (aOR, 5.71; 95% CI, 2.72-11.98) were associated with a greater increase of CHD risk in offspring. Additional adjustments for maternal levels of vitamin B_12_ and homocysteine did not materially change these associations (Table 3). Excluding the participants with missing folate metabolism-related genotypes (eTable 3 in Supplement 1) or using propensity score calculations based on more factors to match non-CHD controls (eTable 4 and eTable 5 in Supplement 1) did not change these findings.

**Figure.**
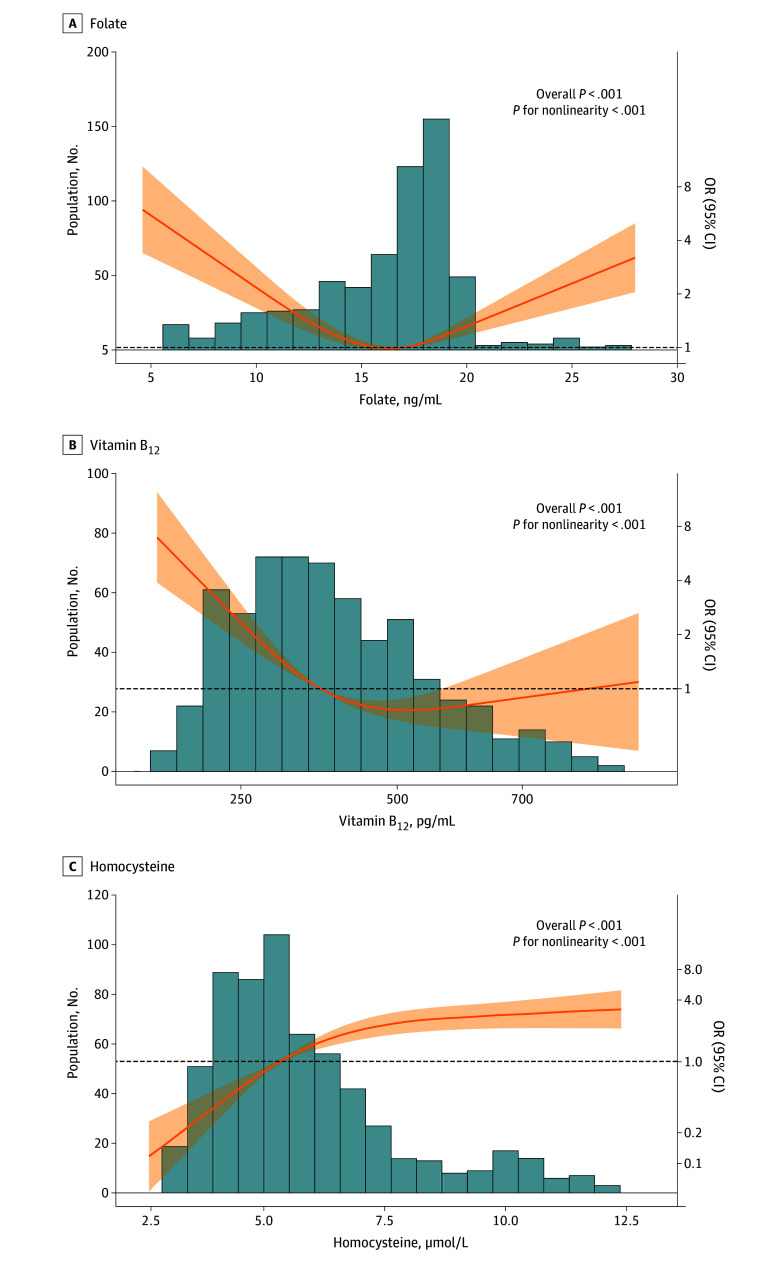
Dose-Response Association of Maternal Levels of Folate, Vitamin B_12_, and Homocysteine With Congenital Heart Disease (CHD) Risk in Offspring The nonlinear associations between maternal serum levels of folate and CHD risk in offspring were examined using conditional logistic regression including a restricted cubic spline term for levels of folate, vitamin B_12_, and homocysteine with adjustment for maternal periconceptional folic acid supplementation, education, occupation, parity, abortion history, pregnancy with diabetes, pregnancy with hypertension, pregnancy with cardiac diseases, infection, in vitro fertilization and embryo transfer, and 5,10-methylenetetrahydrofolate reductase (MTHFR) 677, MTHFR 1298, and 5-methyltetrahydrofolate-homocysteine methyltransferase reductase 66 polymorphisms. Orange lines represent the regression line and shaded areas indicate 95% CIs. OR indicates odds ratio.

**Table 3.** Association Between Maternal Serum Folate Levels and Congenital Heart Disease (CHD) Risk in Offspring

Model^a^	OR (95% CI)		
	Low	Medium	High
Maternal serum levels of folate at early to midpregnancy^b^			
CHD cases/controls, No./No.	51/108	44/284	34/124
Model 1	3.21 (1.98-5.20)	1 [Reference]	1.83 (1.10-3.04)
Model 2	3.05 (1.87-4.98)	1 [Reference]	1.74 (1.05-2.91)
Model 3	3.09 (1.88-5.08)	1 [Reference]	1.81 (1.07-3.06)
Model 3 plus vit B_12_	2.37 (1.41-4.00)	1 [Reference]	2.03 (1.18-3.52)
Model 3 plus HCY	2.17 (1.27-3.71)	1 [Reference]	1.65 (0.96-2.85)
Model 3 plus vit B_12_ plus HCY	1.85 (1.07-3.21)	1 [Reference]	1.80 (1.02-3.18)
WHO criteria^c^			
CHD cases/controls, No./No.	10/4	99/496	20/16
Model 1	20.11 (4.32-93.56)	1 [Reference]	6.00 (2.94-12.21)
Model 2	19.40 (4.00-94.04)	1 [Reference]	5.62 (2.72-11.62)
Model 3	18.97 (3.87-93.11)	1 [Reference]	5.71 (2.72-11.98)
Model 3 plus vit B_12_	12.59 (2.53-62.68)	1 [Reference]	6.51 (2.96-14.34)
Model 3 plus HCY	10.09 (1.91-53.16)	1 [Reference]	4.59 (2.13-9.88)
Model 3 plus vit B_12_ plus HCY	7.61 (1.46-39.81)	1 [Reference]	5.35 (2.39-12.01)

Abbreviations: HCY, homocysteine; OR, odds ratio; vit B_12_, vitamin B_12_; WHO, World Health Organization.

^a^ Model 1 is unadjusted. Model 2 was adjusted for periconceptional folic acid supplementation, maternal education, occupation, parity, abortion history, pregnancy with diabetes, pregnancy with hypertension, pregnancy with cardiac diseases, infection, and in vitro fertilization and embryo transfer. Model 3 was additionally adjusted for maternal 5,10-methylenetetrahydrofolate reductase (MTHFR) 677, MTHFR 1298, and 5-methyltetrahydrofolate-homocysteine methyltransferase reductase 66 polymorphisms.

^b^ For maternal serum levels of folate at early to midpregnancy, low is defined as less than 13.8 ng/mL, medium is defined as 13.8 to 18.5 ng/mL, and high is defined as more than 18.5 ng/mL.

^c^ For WHO criteria, low is defined as less than 5.9 ng/mL, medium is defined as 5.9 to 20 ng/mL, and high is definied as more than 20 ng/mL.

### Interactions Between Maternal Serum Folate Levels and Vitamin B_12_ on CHD Risk in Offspring

We found an L-shaped association between maternal serum levels of vitamin B_12_ and CHD risk in offspring (overall *P* < .001, *P* for nonlinearity <.001) (Figure), suggesting a detrimental association between maternal vitamin B_12_ deficiency and increased risk of CHD in offspring. Compared with the reference group with medium maternal folate and normal vitamin B_12_ levels, the aOR was 1.48 (95% CI, 0.67-3.29) for low folate and normal vitamin B_12_, and increased to 7.30 (95% CI, 3.83-13.89) for low folate and vitamin B_12_ deficiency (Table 4). For individuals with high maternal folate and normal vitamin B_12_ levels, the aOR was 1.54 (95% CI, 0.78-3.10), whereas it increased to 6.51 (95% CI, 2.79-15.20) for high folate and vitamin B_12_ deficiency (Table 4).

**Table 4.** The Joint Association of Maternal Levels of Folate and Vitamin B_12_ and Homocysteine With Congenital Heart Disease (CHD) Risk in Offspring

Maternal serum levels of vitamin B_12_ and homocysteine	Maternal serum levels of folate at early to midpregnancy		
	Low (Q1, <13.8 ng/mL)	Medium (Q2-Q3, 13.8-18.5 ng/mL)	High (Q4, >18.5 ng/mL)
Vitamin B_12_			
Normal^a^			
CHD cases/controls, No.	11/62	29/230	18/98
aOR (95% CI)	1.48 (0.67-3.29)	1 [Reference]	1.54 (0.78-3.10)
Deficiency^b^			
CHD cases/controls, No.	40/46	15/54	16/26
aOR (95% CI)	7.30 (3.83-13.89)	2.18 (1.06-4.52)	6.51 (2.79-15.2)
Multiplicative interaction^c^	*P* = .14	NA	*P* = .25
Homocysteine			
Normal^d^			
CHD case/controls, No.	23/77	30/256	20/104
aOR (95% CI)	2.44 (1.3-4.56)	1 [Reference]	1.59 (0.84-3.01)
Elevated^e^			
CHD case/controls, No.	28/31	14/28	14/20
aOR (95% CI)	8.93 (4.33-18.44)	3.92 (1.80-8.52)	7.17 (2.98-17.27)
Multiplicative interaction^c^	*P* = .90	NA	*P* = .82

Abbreviations: aOR, adjusted odds ratio; NA, not applicable; Q, quantile.

To convert homocysteine to μmol/L, multiply by 7.397; vitamin B_12_ to pmol/L, multiply by 0.7378.

^a^ Normal levels defined as 300 pg/mL and above.

^b^ Deficient levels defined as below 300 pg/mL.

^c^ We used multiplicative interaction analyses with adjustment for maternal 5,10-methylenetetrahydrofolate reductase (MTHFR) 677, MTHFR 1298, and 5-methyltetrahydrofolate-homocysteine methyltransferase reductase 66 polymorphisms, periconceptional folic acid supplementation, education, occupation, parity, abortion history, pregnancy with diabetes, pregnancy with hypertension, pregnancy with cardiac diseases, infection, and in vitro fertilization and embryo transfer.

^d^ Normal levels defined as below 0.95 mg/L.

^e^ Elevated levels defined as 0.95 mg/L and above.

### Interactions Between Maternal Serum Folate Levels and Homocysteine on CHD in Offspring

We found an inverted L-shaped association between maternal serum levels of homocysteine and CHD risk in offspring (overall *P* < .001, *P* for nonlinearity <.001) (Figure), suggesting the detrimental association between maternal elevated homocysteine and increased risk of CHD in offspring. Compared with the reference group with medium maternal folate and normal homocysteine levels, the aOR was 2.44 (95% CI, 1.30-4.56) for low folate and normal homocysteine, and increased to 8.93 (95% CI, 4.33-18.44) for low folate and elevated homocysteine (Table 4). Elevated homocysteine accounted for 32.9% (16.4%-65.0%) of the association between low folate levels and CHD risk (eTable 6 in Supplement 1). Similarly, compared with the reference group, the aOR was 1.59 (95% CI, 0.84-3.01) for high maternal folate and normal homocysteine levels, and increased to 7.17 (95% CI, 2.98-17.27) for high folate and elevated homocysteine (Table 4).

## Discussion

In the current study, we observed a U-shaped association between maternal serum folate levels spot-checked at early to midpregnancy and CHD risk in offspring. Our findings highlighted that besides the association of low maternal serum folate levels during early to midpregnancy with an elevated risk of CHD in offspring, high folate levels may also be associated with an increased risk of CHD. Furthermore, these detrimental associations may be exacerbated in the presence of concurrent maternal vitamin B_12_ deficiency or homocysteine elevation (eFigure 2 in Supplement 1).

The association between folic acid supplementation and CHD was first recognized in the Hungarian randomized controlled trial, which mainly aimed to investigate the effects of folate supplementation on NTDs in the 1980s.^7,31^ This trial indicated that supplementation with multivitamins containing 0.8 mg folic acid from 1 month before conception through at least the second missed menstrual period reduced more than 50% risk of CHD. Since then, conducting placebo-controlled interventional studies to examine the protective effects of folate on CHD was deemed ethically inappropriate. However, subsequent observational studies reported inconsistent findings.^8,9,10,11^ Although meta-analyses of these observational studies showed an association between maternal daily intake of supplements containing at least 0.4 mg folic acid and reduced risk of CHD, approximately half of the individual studies found null results.^8,9,10,11^

Our previous study,^4^ which included 8379 CHD cases and 6918 controls registered in 40 health care centers across 21 administrative areas of Guangdong Province between 2004 and 2016, exhibited a beneficial association between periconceptional folic acid supplementation and a reduced CHD risk. In contrast, our current study, which enrolled 129 cases of CHD and 516 controls from a developed urban area over the period from 2015 to 2018, did not identify a marked association between periconceptional folic acid supplementation and CHD risk. The divergent findings between these studies could be attributed to the substantial difference in the prevalence of folic acid supplementation (12% in our previous study vs 95% in the current study), a shift largely driven by the implementation of China’s National Free Preconception Health Examination Project in 2010 and 2011.^4,32,33^

Despite the associations between folate supplementation and CHD, few studies have examined the association between maternal blood levels of folate during pregnancy and CHD risk. In the current study, we found a U-shaped association between maternal serum folate levels at early to midpregnancy and offspring CHD risk. A recent multicenter nested case-control study^14^ from China, which included 197 CHD cases and 788 individually matched non-CHD controls, found that maternal levels of red blood cell (RBC) folate at early pregnancy were inversely associated with offspring CHD. While both that study and ours identified consistent associations between low maternal folate levels and an elevated risk of CHD in offspring, our findings also revealed that high maternal folate levels were associated with an increased risk of CHD in offspring, a phenomenon not observed in the previous study. This discrepancy could be due to our study assessing serum folate levels, whereas the previous study measured RBC folate levels, as well as our participants having higher folate levels, likely due to higher rates of periconceptional folic acid supplementation, higher educational levels, and lower rates of alcohol consumption (eTable 7 in Supplement 1).

In the current study, we found a significant association between maternal low folate levels and CHD risk in offspring, which was consistent with previous results.^12,13,14^ Moreover, this association was associated with levels of maternal vitamin B_12_ and homocysteine. Specifically, the association between low folate levels and elevated CHD risk was intensified in cases of concurrent vitamin B_12_ deficiency. In contrast, this association was weakened in scenarios of adequate vitamin B_12_ levels or decreased homocysteine levels. The one-carbon metabolism pathway is likely a key mechanism behind the association between low folate levels and CHD risk and explains the observed modification effect of vitamin B_12_ and homocysteine.^34^ Within this pathway, homocysteine is remethylated to form methionine using the methyl from folate with vitamin B_12_ serving as a coenzyme.^20^ Insufficient folate and vitamin B_12_ can lead to increased homocysteine levels,^21^ which is harmful to the cardiovascular system.^35^ Thus, homocysteine might act as a central mediator in the relationships between deficiencies in folate and vitamin B_12_ and the risk of CHD. Additionally, the role of folate extends beyond homocysteine mediation, contributing independently to placental implantation and vascular remodeling, irrespective of vitamin B_12_ and homocysteine levels.^36^

It is noteworthy that we also observed an increased risk of CHD in offspring associated with high maternal folate levels. Furthermore, the co-occurrence of high maternal serum folate levels and vitamin B_12_ deficiency could potentially contribute to an increased number of CHD cases. Conversely, this association was mitigated when accompanied by sufficient vitamin B_12_ levels or reduced homocysteine levels. The mechanisms explaining the relationship between elevated folate levels and CHD risk remain largely unexplored. A recent study^37^ using zebra fish found that excessive folic acid can induce cardiac abnormality by disrupting the methylation level of cardiac marker genes, such as *hand2*, *gata4*, and *nppa*. Furthermore, excessive folic acid intake has been linked to an increased rate of de novo point mutations in DNA.^38^ Vitamin B_12_ deficiency and elevated homocysteine may amplify the association between high folate levels and CHD risk by worsening these pathogenesis pathways, which requires further investigation. Given the rising prevalence of vitamin B_12_ deficiency, often attributed to vegetarian diets, and the subsequent hyperhomocysteinemia,^39^ our study calls for further research to explore the synergistic and complementary effects of vitamin B_12_ and homocysteine on the folate-CHD relationship.

Given the success of folic acid supplementation in reducing NTDs, global recommendations advise women of childbearing age to take at least 0.4 mg of folic acid daily from 1 month before conception through at least 2 months after pregnancy. Meanwhile, about 60 countries have implemented effective mandatory fortification of folic acid in wheat flour, maize flour, or rice.^40^ Together with the increasing awareness of the importance of folate in the general population, an increasing portion of pregnant women take folic acid exceeding the recommended daily allowance of 0.4 mg or even the tolerable upper intake level of 1 mg per day.^18,19^ Our findings of a potential U-shaped association between maternal folate levels during pregnancy and offspring CHD risk highlighted that excessive folate may not confer additional benefits and could potentially have adverse effects. Thus, the previous one-size-fits-all model of folate supplementation and fortification should be reconsidered with caution.

### Strengths and Limitations

The strengths of our study included the large sample size in the CHD research field, the thorough algorithm for the identification of CHD cases, the consideration of maternal genetic polymorphisms affecting folate metabolism in the analyses, the examination of the synergistic impact of maternal folate, vitamin B_12_, and homocysteine on CHD, and the adoption of both the quartile and the WHO criteria for classifying the serum folate levels to ensure the robustness of our findings. However, several limitations should be considered when interpreting our results. First, while both serum and RBC folate levels assess folate deficiency, they provide different insights. Serum folate identifies recent changes in intake, whereas RBC folate indicates chronic status. Therefore, our findings reflect short-term folate status. Second, maternal serum levels of folate were measured at a single time point (16-18 weeks of gestation), making it difficult to relate these levels to preconception and early postconception periods. Third, serum folate levels can be influenced by factors such as supplementation, diet, genetics, and metabolism. Unfortunately, we did not collect dietary intake data, and therefore cannot account for the related bias. Fourth, although our sample size is relatively large for CHD research, the stratified analysis may lack sufficient power to detect interactions adequately. Fifth, paternal characteristics and folate levels during periconceptional periods were not considered in the current study. Sixth, our findings may be subjected to residual confounding due to the observational study design. However, it is ethically challenging to conduct a randomized clinical trial to make causal inferences. Seventh, The WHO classification for macrocytic anemia may not suit pregnant women, as folate levels typically decline during pregnancy.^41^ However, since there is no established serum folate threshold for women of reproductive age, it remains the only classification for validating our findings on both low and high maternal folate levels and CHD risk. Additionally, all participants were recruited from a top cardiac referral center in Southern China, which may limit the generalizability of our findings to other populations.

## Conclusions

Our study revealed that low maternal serum folate levels during early to midpregnancy were associated with a higher risk of CHD in offspring. Intriguingly, it also suggested that excessively high folate levels could be associated with an increased CHD risk. Additionally, these associations might be modulated by vitamin B_12_ and homocysteine levels, though the underlying mechanisms have yet to be investigated. These findings support the notion that pregnant women could benefit most from a tailored and precise folic acid supplementation strategy.

## Notes

Supplement 1.
eMethods
**eFigure 1. **Flow Chart of the Study Participants' Selection **eFigure 2. **Maternal Serum Levels of Folate at Early to Midpregnancy and Risk of Congenital Heart Diseases (CHD) in Offspring **eTable 1. **Maternal Serum Folate Levels According to Folate Metabolism-Related Genetic Polymorphisms **eTable 2. **Maternal Serum Folate Levels According to Periconceptional Folic Acid Supplementation **eTable 3. **Association Between Maternal Serum Folate Levels and CHD Risk in Offspring, Sensitivity Analysis by Excluding 46 Participants With Missing Folate Metabolism-Related Genetic Polymorphisms **eTable 4. **Maternal Characteristics According to offspring’s Congenital Heart Disease Status, Sensitivity Analysis Using Propensity Score Matching on Maternal Age, Education, Occupation, Parity, and Folic Acid Supplementation at a Ratio of 1:4 **eTable 5. **Association between Maternal Serum Folate Levels and CHD Risk in Offspring, Sensitivity Analysis Using Propensity Score Matching on Maternal Age, Education, Occupation, Parity, and Folic Acid Supplementation at a Ratio of 1:4 **eTable 6. **Mediating Effects of Maternal Elevated Homocysteine on the Association of Low Folate and Vitamin B_12_ Deficiency With Congenital Heart Disease Risk in Offspring **eTable 7. **Comparison of Maternal Serum Folate Levels Between Our Study and Chen’s Study

Supplement 2.
Data Sharing Statement
