# Supplementary material for: Maternal Serum Folate During Pregnancy and Congenital Heart Disease in Offspring
Source: JAMA Netw Open. 2024 Oct 10;7(10):e2438747. doi: 10.1001/jamanetworkopen.2024.38747 (PMC11581582; doi:10.1001/jamanetworkopen.2024.38747)
Supplement: Supplement 1. — eMethods eFigure 1. Flow Chart of the Study Participants' Selection eFigure 2. Maternal Serum Levels of Folate at Early to Midpregnancy and Risk of Congenital Heart Diseases (CHD) in Offspring eTable 1. Maternal Serum Folate Levels According to Folate Metabolism-Related Genetic Polymorphisms eTable 2. Maternal Serum Folate Levels According to Periconceptional Folic Acid Supplementation eTable 3. Association Between Maternal Serum Folate Levels and CHD Risk in Offspring, Sensitivity Analysis by Excluding 46 Participants With Missing Folate Metabolism-Related Genetic Polymorphisms eTable 4. Maternal Characteristics According to offspring’s Congenital Heart Disease Status, Sensitivity Analysis Using Propensity Score Matching on Maternal Age, Education, Occupation, Parity, and Folic Acid Supplementation at a Ratio of 1:4 eTable 5. Association between Maternal Serum Folate Levels and CHD Risk in Offspring, Sensitivity Analysis Using Propensity Score Matching on Maternal Age, Education, Occupation, Parity, and Folic Acid Supplementation at a Ratio of 1:4 eTable 6. Mediating Effects of Maternal Elevated Homocysteine on the Association of Low Folate and Vitamin B12 Deficiency With Congenital Heart Disease Risk in Offspring eTable 7. Comparison of Maternal Serum Folate Levels Between Our Study and Chen’s Study [file jamanetwopen-e2438747-s001.pdf]

Supplemental Online Content

Qu Y, Liu X, Lin S, et al. Maternal serum folate during pregnancy and congenital heart disease in offspring. *JAMA Netw Open*. 2024;7(10):e2438747. doi:10.1001/jamanetworkopen.2024.38747

eMethods

eFigure 1. Flow Chart of the Study Participants' Selection

eFigure 2. Maternal Serum Levels of Folate at Early to Midpregnancy and Risk of Congenital Heart Diseases (CHD) in Offspring

eTable 1. Maternal Serum Folate Levels According to Folate Metabolism-Related Genetic Polymorphisms

eTable 2. Maternal Serum Folate Levels According to Periconceptional Folic Acid Supplementation

eTable 3. Association Between Maternal Serum Folate Levels and CHD Risk in Offspring, Sensitivity Analysis by Excluding 46 Participants With Missing Folate Metabolism-Related Genetic Polymorphisms

eTable 4. Maternal Characteristics According to offspring's Congenital Heart Disease Status, Sensitivity Analysis Using Propensity Score Matching on Maternal Age, Education, Occupation, Parity, and Folic Acid Supplementation at a Ratio of 1:4

eTable 5. Association between Maternal Serum Folate Levels and CHD Risk in Offspring, Sensitivity Analysis Using Propensity Score Matching on Maternal Age, Education, Occupation, Parity, and Folic Acid Supplementation at a Ratio of 1:4

eTable 6. Mediating Effects of Maternal Elevated Homocysteine on the Association of Low Folate and Vitamin B_{12} Deficiency With Congenital Heart Disease Risk in Offspring

eTable 7. Comparison of Maternal Serum Folate Levels Between Our Study and Chen's Study

This supplemental material has been provided by the authors to give readers additional information about their work.

© 2024 Qu Y et al. *JAMA Network Open.*

© 2024 Qu Y et al. *JAMA Network Open.*

eMethods

CHD diagnosis

CHD was diagnosed and confirmed through a comprehensive multi-stage approach, which involved fetal ultrasound screening, fetal echocardiography examination, neonatal cardiac evaluations, postnatal echocardiography confirmation, and follow-up assessments of the infants. In detail, all pregnancies underwent basic ultrasound screening for fetal cardiac anomalies between 18 and 24 weeks of gestation. Suspected CHD fetuses were further evaluated through echocardiography at 22 to 26 weeks of gestation to confirm the diagnosis. Following delivery, each newborn received a clinical cardiac assessment before discharge, usually within 72 hours, according to international guidelines. Newborns with suspected CHD and those prenatally diagnosed underwent postnatal echocardiography to confirm the CHD diagnoses. Live births were followed up until one year after birth to identify any late-identifying CHD. CHD diagnoses were further corroborated through additional methods, such as autopsy, cardiac catheterization, and surgery when available. Each CHD case was reviewed by two senior pediatric cardiologists, and any disagreements were resolved by a third one. One predominant phenotype with the most severe hemodynamic pathology was assigned to each case with multiple CHD diagnoses. A total of 172 infants were diagnosed with CHD among these pregnancies.

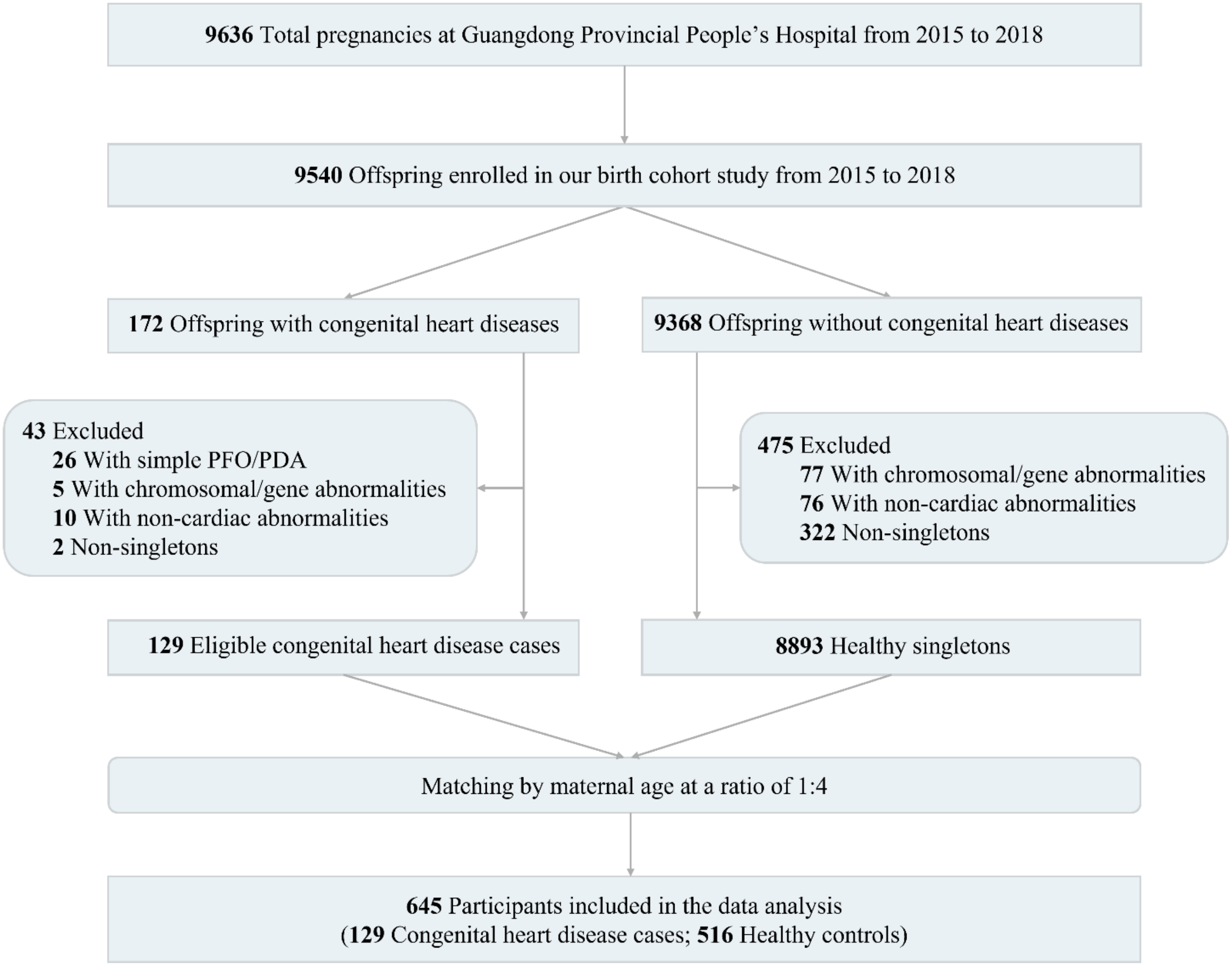

eFigure 1. Flow Chart of the Study Participants' Selection

Abbreviations: PDA, patent ductus arteriosus; PFO, patent foramen ovale.

© 2024 Qu Y et al. *JAMA Network Open.*

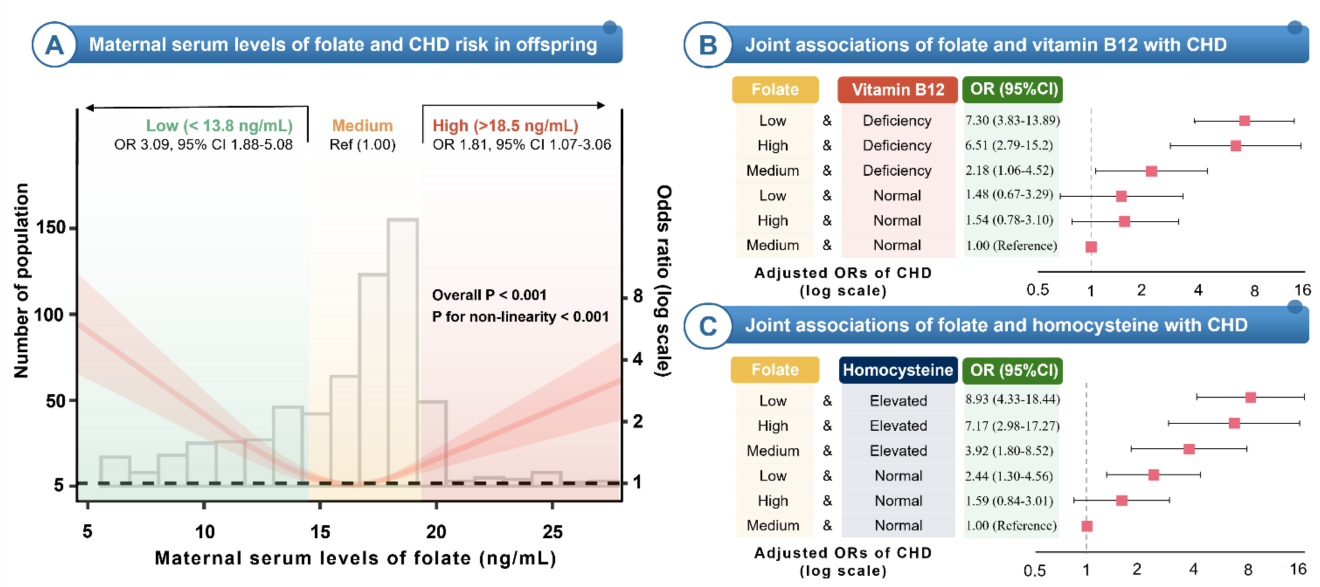

eFigure 2. Maternal Serum Levels of Folate at Early to Midpregnancy and Risk of Congenital Heart Diseases (CHD) in Offspring

© 2024 Qu Y et al. *JAMA Network Open.*

eTable 1. Maternal Serum Folate Levels According to Folate Metabolism-Related Genetic Polymorphisms

	Maternal folate metabolism-related genetic polymorphisms			
**MTHFR 677**	**CC**	**CT**	**TT**	***P* value**
Total No.	329	216	54	
CHD cases, No. (%)	74 (22.5)	35 (16.2)	13 (24.1)	0.16
Maternal serum folate levels (ng/mL)	17.00 (13.6-18.5)	17.25 (14.4-18.6)	17.1 (12.6-18.4)	0.76
Quartiles of maternal serum folate levels				
Low (Q1, <13.8 ng/mL)	88 (26.7)	45 (20.8)	14 (25.9)	
Medium (Q2-Q3, 13.8-18.5 ng/mL)	159 (48.3)	117 (54.2)	29 (53.7)	0.51
High (Q4, >18.5 ng/mL)	82 (24.9)	54 (25.0)	11 (20.4)	
**MTHFR 1298**	**AA**	**AC**	**CC**	***P* value**
Total No.	353	216	30	
CHD cases, No. (%)	78 (22.1)	37 (17.1)	7 (23.3)	0.33
Maternal serum folate levels (ng/mL)	16.9 (13.6-18.5)	17.2 (14.1-18.6)	17.1 (15.7-18.5)	0.56
Quartiles of maternal serum folate levels				
Low (Q1, <13.8 ng/mL)	92 (26.1)	50 (23.1)	5 (16.7)	
Medium (Q2-Q3, 13.8-18.5 ng/mL)	179 (50.7)	108 (50.0)	18 (60.0)	0.64
High (Q4, >18.5 ng/mL)	82 (23.2)	58 (26.9)	7 (23.3)	
**MTRR 66**	**AA**	**AG**	**GG**	***P* value**
Total No.	331	216	52	
CHD cases, No. (%)	66 (19.9)	48 (22.2)	8 (15.4)	0.52
Maternal serum folate levels (ng/mL)	17.2 (14.0-18.5)	17.1 (13.9-18.6)	16.4 (12.9-18.6)	0.52
Quartiles of maternal serum folate levels				
Low (Q1, <13.8 ng/mL)	77 (23.3)	52 (24.1)	18 (34.6)	
Medium (Q2-Q3, 13.8-18.5 ng/mL)	174 (52.6)	110 (50.9)	21 (40.4)	0.44
High (Q4, >18.5 ng/mL)	80 (24.2)	54 (25.0)	13 (25.0)	

Abbreviation: CHD, congenital heart disease; MTHFR, 5,10-methylenetetrahydrofolate reductase; MTRR, 5-methyltetrahydrofolate-homocysteine methyltransferase reductase; Q, quartile.

© 2024 Qu Y et al. *JAMA Network Open.*

eTable 2. Maternal Serum Folate Levels According to Periconceptional Folic Acid Supplementation

	**Maternal periconceptional folic acid supplementation**		
	**Yes**	**No**	***P* value**
Total No.	611	34	
CHD cases, No. (%)	122 (20.0)	7 (20.6)	0.93
Maternal serum folate levels (ng/mL)	17.1 (14.0-18.6)	14.7 (11.4-17.8)	0.001
Quartiles of maternal serum folate levels			
Low (Q1, <13.8 ng/mL)	142 (23.2%)	17 (50.0%)	
Medium (Q2-Q3, 13.8-18.5 ng/mL)	314 (51.4%)	14 (41.2%)	0.01
High (Q4, >18.5 ng/mL)	155 (25.4%)	3 (8.8%)	

Abbreviation: CHD, congenital heart disease; Q, quartile

eTable 3. Association Between Maternal Serum Folate Levels and CHD Risk in Offspring, Sensitivity Analysis by Excluding 46 Participants With Missing Folate Metabolism-Related Genetic Polymorphisms

	**Maternal serum levels of folate at early-mid pregnancy**		
	**Low (Q1, <13.8 ng/mL)**	**Medium (Q2-Q3, 13.8-18.5 ng/mL)**	**High (Q4, >18.5 ng/mL)**
CHD cases/Controls	48/99	42/263	32/115
ORs (95% CI)			
Model 1	3.21 (1.98-5.20)	Ref	1.83 (1.10-3.04)
Model 2	3.05 (1.87-4.98)	Ref	1.74 (1.05-2.91)
Model 3	3.20 (1.90-5.39)	Ref	1.84 (1.07-3.16)
Model 3 + Vit B12	2.45 (1.42-4.24)	Ref	2.00 (1.14-3.51)
Model 3 + Hcy	2.12 (1.21-3.71)	Ref	1.69 (0.97-2.94)
Model 3 + Vit B12 + Hcy	1.77 (0.99-3.15)	Ref	1.80 (1.01-3.22)
		**WHO criteria**	
	**Deficiency (<5.9 ng/mL)**	**Normal (5.9-20 ng/mL)**	**Elevated (>20 ng/mL)**
CHD cases/Controls	10/4	93/457	19/16
ORs (95% CI)			
Model 1	20.11 (4.32-93.56)	Ref	6.00 (2.94-12.21)
Model 2	19.40 (4.00-94.04)	Ref	5.62 (2.72-11.62)
Model 3	16.77 (3.38-83.11)	Ref	5.26 (2.49-11.10)
Model 3 + Vit B12	11.03 (2.18-55.92)	Ref	6.10 (2.70-13.79)
Model 3 + Hcy	9.22 (1.75-48.53)	Ref	4.10 (1.88-8.95)
Model 3 + Vit B12 + Hcy	6.69 (1.27-35.24)	Ref	4.85 (2.08-11.30)

Model 1 is unadjusted. Model 2 was adjusted for periconceptional folic acid supplementation, maternal education, occupation, parity, abortion history, pregnancy with diabetes, pregnancy with hypertension, pregnancy with cardiac diseases, infection, and in vitro fertilization and embryo transfer. Model 3 was additionally adjusted for maternal MTHFR 677, MTHFR 1298, and MTRR 66 polymorphisms.

Abbreviations: CHD, congenital heart disease; CI, confidential interval; Hcy, homocysteine; OR, odds ratio; Q, quartile; Ref, reference; Vit B12, vitamin B12.

© 2024 Qu Y et al. *JAMA Network Open.*

eTable 4. Maternal Characteristics According to offspring's Congenital Heart Disease Status, Sensitivity Analysis Using Propensity Score Matching on Maternal Age, Education, Occupation, Parity, and Folic Acid Supplementation at a Ratio of 1:4

	**CHD cases (N=129)**	**Controls (N=516)**	***P-value***
**Matching characteristics**			
Age (years)	31.5±5.45	31.6±5.25	0.93
Education (years)			
≤12	3 (2.3)	9 (1.7)	0.66
>12	126 (97.7)	507 (98.3)	
Unemployed			
Yes	16 (12.4)	71 (13.8)	0.68
No	113 (87.6)	445 (86.2)	
Nulliparous			
Yes	72 (55.8)	287 (55.6)	0.97
No	57 (44.2)	229 (44.4)	
Periconceptional folic acid supplementation			
Yes	122 (94.6)	482 (93.4)	0.63
No	7 (5.4)	34 (6.6)	
**Gestational weeks at blood collection**	16.45±5.57	16.85±2.55	0.51
**Serum folate** (ng/mL)	16.0 (10.6-18.6)	17.3 (14.7-18.5)	0.06
**Serum vitamin B12** (pg/mL)	280 (208-399)	391 (305-507)	<0.001
**Serum homocysteine** (μmol/L)	6.7 (5.3-8.8)	5.3 (4.4-6.3)	<0.001
**Folate metabolism-related gene polymorphisms**			
MTHFR 677			
CC	74 (60.7)	287 (59.1)	0.38
CT	35 (28.7)	166 (34.2)	
TT	13 (10.7)	33 (6.8)	
MTHFR 1298			
AA	78 (63.9)	284 (58.4)	0.45
AC	37 (30.3)	175 (36.0)	
CC	7 (5.7)	27 (5.6)	
MTRR 66			
AA	66 (54.1)	277 (57.0)	0.73
AG	48 (39.3)	176 (36.2)	
GG	8 (6.6)	33 (6.8)	

© 2024 Qu Y et al. *JAMA Network Open.*

**Other characteristics**			
Elective/spontaneous abortion history			
Yes	53 (41.1)	194 (37.6)	0.47
No	76 (58.9)	322 (62.4)	
Prepregnant BMI (kg/m^2)	20.7±3.0	21.2±3.2	0.18
Pregnancy with diabetes			
Yes	18 (14.0)	96 (18.6)	0.22
No	111 (86.0)	420 (81.4)	
Pregnancy with hypertension			
Yes	10 (7.8)	28 (5.4)	0.32
No	119 (92.2)	488 (94.6)	
Pregnancy with cardiac disease			
Yes	15 (11.6)	45 (8.7)	0.32
No	114 (88.4)	471 (91.3)	
Pregnancy with infection			
Yes	16 (12.4)	54 (10.5)	0.53
No	113 (87.6)	462 (89.5)	
IVF-ET			
Yes	6 (4.7)	28 (5.4)	0.72
No	123 (95.3)	488 (94.6)	
Antibiotics use			
Yes	7 (5.4)	39 (7.6)	0.40
No	122 (94.6)	477 (92.4)	
Anti-miscarriage medicine uses			
Yes	11 (8.5)	45 (8.7)	0.92
No	118 (91.5)	471 (91.3)	

Continuous variables are described as mean ± SD for those with normal distribution and median (IQR) for those with nonnormal distribution. Categorical variables were presented as numbers (percent). The comparison of maternal characteristics between CHD cases and non-CHD controls was conducted using univariate conditional logistic regression.

Abbreviations: BMI, body mass index; CHD, congenital heart disease; IVF-ET, *in vitro* fertilization and embryo transfer; MTHFR, 5,10-methylenetetrahydrofolate reductase; MTRR, 5-methyltetrahydrofolate-homocysteine methyltransferase reductase.

© 2024 Qu Y et al. *JAMA Network Open.*

eTable 5. Association between Maternal Serum Folate Levels and CHD Risk in Offspring, Sensitivity Analysis Using Propensity Score Matching on Maternal Age, Education, Occupation, Parity, and Folic Acid Supplementation at a Ratio of 1:4

	**Maternal serum levels of folate at early-mid pregnancy**		
	**Low (Q1, <13.8 ng/mL)**	**Medium (Q2-Q3, 13.8-18.5 ng/mL)**	**High (Q4, >18.5 ng/mL)**
CHD cases/Controls	51/101	44/288	34/127
ORs (95% CI)			
Model 1	3.30 (2.07-5.26)	Ref	1.67 (1.03-2.74)
Model 2	3.34 (2.08-5.36)	Ref	1.65 (1.01-2.71)
Model 3	3.24 (2.00-5.24)	Ref	1.67 (1.02-2.74)
Model 3 + Vit B12	2.46 (1.48-4.08)	Ref	1.87 (1.13-3.11)
Model 3 + Hcy	2.13 (1.27-3.58)	Ref	1.59 (0.96-2.64)
Model 3 + Vit B12 + Hcy	1.80 (1.06-3.07)	Ref	1.75 (1.04-2.93)
	**WHO criteria**		
	**Deficiency (<5.9 ng/mL)**	**Normal (5.9-20 ng/mL)**	**Elevated (>20 ng/mL)**
CHD cases/Controls	10/5	99/505	20/6
ORs (95% CI)			
Model 1	10.84 (3.28-35.82)	Ref	16.62 (6.16-44.79)
Model 2	10.78 (3.21-36.25)	Ref	16.52 (6.12-44.58)
Model 3	10.85 (3.13-37.60)	Ref	17.01 (6.22-46.53)
Model 3 + Vit B12	6.76 (1.91-23.95)	Ref	15.69 (5.58-44.11)
Model 3 + Hcy	7.50 (2.00-28.13)	Ref	17.39 (6.15-49.21)
Model 3 + Vit B12 + Hcy	5.50 (1.45-20.86)	Ref	16.04 (5.59-46.02)

Model 1 is unadjusted. Model 2 was adjusted for abortion history, pregnancy with diabetes, pregnancy with hypertension, pregnancy with cardiac diseases, infection, and in vitro fertilization and embryo transfer. Model 3 was additionally adjusted for maternal MTHFR 677, MTHFR 1298, and MTRR 66 polymorphisms.

Abbreviations: CHD, congenital heart disease; CI, confidential interval; Hcy, homocysteine; OR, odds ratio; Q, quartile; Ref, reference; Vit B12, vitamin B12.

© 2024 Qu Y et al. *JAMA Network Open.*

eTable 6. Mediating Effects of Maternal Elevated Homocysteine on the Association of Low Folate and Vitamin B_{12} Deficiency With Congenital Heart Disease Risk in Offspring

	Indirect effect	Direct effect	Total effect	Proportion mediated (%)	*P* value
Low folate	0.05 (0.03-0.08)	0.11 (0.03-0.20)	0.16 (0.08-0.25)	32.9 (16.4-65.0)	<0.001
Vitamin B12 deficiency	0.04 (0.02-0.06)	0.17 (0.10-0.25)	0.21 (0.14-0.29)	18.4 (10.2-31.0)	<0.001

The covariates adjusted for in the mediation analyses included maternal MTHFR 677, MTHFR 1298, MTRR 66 polymorphisms, periconceptional folic acid supplementation, maternal education, occupation, parity, abortion history, pregnancy with diabetes, pregnancy with hypertension, pregnancy with cardiac diseases, infection, and *in vitro* fertilization and embryo transfer.

eTable 7. Comparison of Maternal Serum Folate Levels Between Our Study and Chen's Study

Maternal serum folate levels, Median (Inter-Quartile Range)	Our study		Chen's study	
	CHD cases	Non-CHD controls	CHD cases	Non-CHD controls
Unit: ng/mL	16.0 (10.6-18.6)	17.2 (14.4-18.5)		
Unit: nmol/L[a]	36.3 (24.1-42.2)	39.0 (32.7-42.0)	31 (21-38)	32 (21-39)

[a] To convert the folate values from ng/mL, multiply 2.265 (Chen et al., 2022).

Abbreviations: CHD, congenital heart disease.

© 2024 Qu Y et al. *JAMA Network Open.*
