# Supplementary material for: Maternal Serum Folate During Pregnancy and Congenital Heart Disease in Offspring
Source: JAMA Netw Open. 2024 Oct 10;7(10):e2438747. doi: 10.1001/jamanetworkopen.2024.38747 (PMC11581582; doi:10.1001/jamanetworkopen.2024.38747)
Supplement: Supplement 2. — Data Sharing Statement [file jamanetwopen-e2438747-s002.pdf]

Data Sharing Statement

Qu. Maternal Serum Folate During Pregnancy and Congenital Heart Disease in Offspring. *JAMA Netw Open*. Published October 10, 2024. doi:10.1001/jamanetworkopen.2024.38747

Data
Data available: Yes
Data types: Deidentified participant data
How to access data: chenjimei@gdph.org.cn
When available: With publication

Supporting Documents
Document types: None

Additional Information
Who can access the data: researchers whose proposed use of the data has been approved
Types of analyses: for a specified purpose
Mechanisms of data availability: after approval of a proposal and with a signed data access agreement
